# Determining the Levels of Volatile Organic Pollutants in Urban Air Using a Gas Chromatography-Mass Spectrometry Method

**DOI:** 10.1155/2009/148527

**Published:** 2010-02-03

**Authors:** Simona Nicoara, Loris Tonidandel, Pietro Traldi, Jonathan Watson, Geraint Morgan, Ovidiu Popa

**Affiliations:** ^1^Technical University, 15 C. Daicoviciu Street, 400020 Cluj-Napoca, Romania; ^2^Planetary and Space Sciences Research Institute, The Open University, Milton Keynes MK7 6AA, UK; ^3^Mass Spectrometry Laboratory, Institute for Molecular Science and Technology, CNR, Corso Stati Uniti 4, Padova, Italy; ^4^Centre for Environment and Health, 23A Cetatii Street, 400 166 Cluj-Napoca, Romania

## Abstract

The paper presents the application of a method based on coupled gas chromatography-mass spectrometry, using an isotopically labelled internal standard for the quantitative analysis of benzene (B), toluene (T), ethyl benzene (E), and o-, m-, p-xylenes (X). Their atmospheric concentrations were determined based on short-term sampling, in different sites of Cluj-Napoca, a highly populated urban centre in N-W Romania, with numerous and diversified road vehicles with internal combustion engines. The method is relatively inexpensive and simple and shows good precision and linearity in the ranges of 7–60 *μ*g/m^3^ (B), 13–90 *μ*g/m^3^ (T), 7–50 *μ*g/m^3^ (E), 10–70 *μ*g/m^3^ (X-m,p), and 20–130 *μ*g/m^3^ (X-o). The limits of quantitation/detection of the method LOQ/LOD are of 10/5 *μ*g/m^3^ (Xo), 5/3 *μ*g/m^3^ (B, E, X-m,p), and of 3/1 *μ*g/m^3^ (T), respectively.

## 1. Introduction

Even when present at low concentrations compared to other air contaminants, due to their toxic properties VOCs are of concern as they pose risk for humans health [1, 2] and for the environment [3–5]. Of the volatile atmospheric contaminants, benzene, toluene, ethyl-benzene, and xylenes, shortly named BTEX, mainly originate from exhaust gases of internal combustion vehicles and are of particular concern in urban areas with intense road traffic, hence the interest for monitoring their atmospheric concentrations [4, 6–8]. 

 Methods for determining atmospheric levels of VOCs use preconcentration from canister samples [9] or passive/active trapping on solid adsorbents [1, 6, 9]. To avoid the use of a cryogenic trap prior to injection onto the GC column, and based on our previous results on analysing VOCs from laboratory air [10], charcoal was the preferred adsorbent instead of TenaxTA. The BTEX analytes are then desorbed and separated on the GC column, the GC-MS coupling [1, 6, 9–16] providing high specificity and sensitivity in the detection of atmospheric volatile contaminants and particulate matter [16]. 

 The aim of this study is to apply an SIM/GC-MS method for determining the levels of atmospheric BTEX concentrations. The methods high selectivity and specificity are due to the mass spectrometer operating in the selected ion monitoring mode SIM, which allowed the peak deconvolution of benzene and its perdeuterated analogue internal standard, as they were coeluting in the total ion chromatogram. 

## 2. Experimental

Homemade calibration solutions of the BTEX analytes (benzene, toluene, ethyl benzene, m-, p-, and o-xylene) were prepared, with concentrations ranging between 40 and 500 pL/mL, in dichloromethane DCM (Fluka, p. a.). The solvent, used in previous studies [10], elutes early without interfering with the analytes. In each calibration solution, the internal standard, perdeuterated benzene from Supelco, was added in a constant concentration of 2 nL/mL. All the compounds used were of analytical grade purity. Of each calibration solution, 1 microlitre was injected into the GC.

### 2.1. Sampling and Desorption of Analytes

Preconcentration of the atmospheric contaminants was performed in SKC glass tubes filled with charcoal, using a portable, battery-operated pump set at 100 mL/min air flow, 45-minutes sampling time. A manually operated mechanical counter was used to determine the number of petrol-fueled vehicles passing the sampling site. The air samples were collected between 17:00 and 20:00 during week days, in the cold season. The analytes were then desorbed in 0.5 mL dichloromethane DCM, with 1-minute vortex and 2-minute ultrasonication. An internal standard IS, deuterated benzene, was added to result in 2 nL/mL concentration in each sample extract, and 1 microlitre of the supernatant was injected in the GC.

### 2.2. Equipment

The analytes were separated using an Agilent GC 6890 gas chromatograph equipped with a capillary column DB-5 (30 m × 0.25 mm × 0.25 *μ*m), with the GC oven temperature program: 30°C (5′) to 150°C at 7°C/min, then to 270°C, at 20°C/min . The injector temperature was 270°C, with a split 10 : 1 and He carrier gas at 1.1 mL/min flow. The mass spectrometer Agilent model 5973 is operated in the selected ion monitoring mode SIM, under standard conditions, with 70 eV ionization energy, 230°C EI ion source temperature, and the quadrupolar mass detector at 150°C. 

## 3. Results and Discussion

The GC-MS response, averaged for duplicate injections, was calibrated using the ratio of the selected ions peak areas in each analyte to the perdeuterated benzene peak area at m/z 82 Da. 

**Figure 1:**
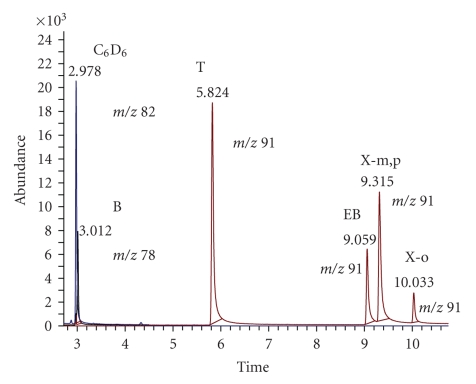
Example chromatogram of sample extract #1: separation of deuterated benzene (IS) and of the BTEX analytes on capillary column DB-5 30 m× 0.25 mm × 0.25 *μ*m. Temperature: 30°C (5′) to 150°C at 7°C/min, then to 270°C, at 20°C/min.

Figure 1 shows an example chromatogram of the extracted ions at m/z 78 (benzene, B), m/z 82 (deuterated benzene, C_6_D_6_), and m/z 91 (toluene T, ethyl-benzene E, m,p-Xylene X_m,p_; o-xylene X_o_) from the total ion chromatogram, of the air sample collected at site #1, on Horea street. 

The calibration equations and the method characteristics for each compound are shown in Table 1. Possible losses during and after collection were reduced by immediately recapping the sampling tubes and storage at 4°C prior to analysis. The extraction yield from charcoal was enhanced by extending the vortex time and by including the ultrasonication extraction step. The total recovery values depended on the analyte: 55% (B), 36% (T); 72% (EB), 48% (m,p-X), and 25% (X-o), respectively.

The method shows good precision (*n* = 6) with RSD values between 4.7% (benzene) and 13.2% (X-m,p), while accuracy RSD was between 9.7% (T) and 13.1% (X-o). The limits of quantization/detection of the method LOQ/LOD are 3/1 *μ*g/m^3^ (T), 5/3 *μ*g/m^3^ (B, E, X-m, p), and 10/5 *μ*g/m^3^ air (X-o). 

Table 2 presents the atmospheric concentrations of BTEX found at the 9 monitored sites. Their values range between 7 and 22 *μ*g/m^3^ (B), 18–72 *μ*g/m^3^ (T), 7–23 *μ*g/m^3^ (E), 10–61 *μ*g/m^3^ (X-m, -p), and 23–40 *μ*g/m^3^ of air (X-o). The highest BTEX levels in air were found on Horea street (samples #1–3), and the average number of petrol fuelled vehicles ranged between *N* = 15–25/min during sampling. The road has double lanes in two ways and connects all the major routes to the city Railway Station and to the North exit. The highest BTEX air concentrations were found at site #1 (17:30 hours), a very busy street junction, and were only slightly decreasing at sites #2 (middle of the street; 18:30 hours) and #3 (the street end to the Railway Station square; 20:00 hours), the evening decreasing trend being similar to those observed in comparable European towns [14, 15].

Aurel Vlaicu street (samples #7–9) is a four-lane dual carriage way and represents the main access to the S-E exit of the town. It supports heavy trucks, and busy traffic, being the widest of the city boulevards. Despite a high average of 38 internal combustion vehicles per minute during sampling, the atmospheric concentrations of volatiles were smaller than those found on Horea street, due to the lower temperatures and light rain during sampling at site #7–9.

The concentrations of BTEX in the Central Park zone, samples #4–6, were the lowest, while the traffic along the park limit slightly decreased in time, between 17:00 and 20:00 sampling hours. 

In sampling sites #1–3 and #7–9, both roads cross residential areas exposed to outdoor air polluted from road traffic. Among BTEX, toluene and xylenes (m,p) were found to be the major contributors to air contamination from engine exhausts gas. At sites 1–3 and 7–9, that is, on busy streets and street crossings, short-term sampling concentrations found for benzene in air exceeded 10 *μ*g/m^3^, the recommended annual average limit [11]. The ratio of toluene to benzene concentration ranges between 1.6 and 6, comparable to values found in similar studies [8, 14, 15]. BTEX concentrations in urban atmosphere show a wide variability, caused by specific conditions, including the dimension and technical state of the vehicle fleet, the road traffic intensity, and meteorological factors. The summer atmospheric concentrations of BTEX in air are expected to be higher [3, 9, 17] over longer time intervals, due to an increase of air temperature and of road traffic.

## 4. Conclusions

A chromatographic-mass spectrometric method was prepared and applied to assess atmospheric concentrations of BTEX in an urban centre of Romania, with a high density of vehicles with internal combustion. The GC-MS method has good precision and accuracy in the required dynamic range. In all the sites, the short-term sampling concentration of benzene in air may exceed the annual average value aimed as limit, while toluene and xylenes (m,p) are the most abundant of the BTEX air contaminants in the urban environment monitored.

## Figures and Tables

**Table 1:** Compounds analyzed and method characteristics: calibration equations and coefficients of correlation, linearity range, limits of quantization and of detection, precision, and accuracy RSD (*n* = 6).

Compound	Equation	*R* ^2^	Linearity range (*μ*g/m^3^)	LOQ (*μ*g/m^3^)	LOD (*μ*g/m^3^)	Precision RSD (%)	Accuracy RSD (%)
Benzene	*y* = 0.0026 × −0.00569	0.974	7–60	5	3	4.7	10.1
Toluene	*y* = 0.0055 × +0.00903	0.964	13–90	3	1	11.2	9.7
Ethylbenzene	*y* = 0.0031 × −0.1593	0.979	7–50	5	3	11.1	12.3
m-, p-Xylenes	*y* = 0.0050 × −0.3645	0.946	10–70	5	3	13.0	11.6
o-Xylene	*y* = 0.0069 × −0.4757	0.965	20–130	10	5	13.1	13.1

**Table 2:** Values of the BTEX concentrations found in street air, during winter season, between: 17:00 and 20:00. *N*_*av*_: average number of vehicles per minute, during sampling. Analytes: B (benzene), T (toluene), E (ethyl-benzene), X m, p, o (xylenes) T/B: toluene/benzene ratio.

Crt. Nr.	Sampling site		*N*_av_Veh/min	B *μ*g/m^3^	T *μ*g/m^3^	E *μ*g/m^3^	m,p-X *μ*g/m^3^	o-X *μ*g/m^3^	T/B
1			25	21.4	71.9	22.7	61.4	39.6	3.4
2	Horea street		26	21.2	64.1	19.4	45.6	33.4	3.0
3			15	22.2	62.2	18.6	50.0	35.2	2.8

4		Limit		8.8	53.9	8.5	15.8	27.4	6.1
5	Central Park	10 m inside	19	7.8	42.72	8.2	12.1	26.6	5.4
6		50 m inside		7.7	31.9	6.7	9.9	23.4	4.1

7			30	10.5	56.0	11.7	16.2	35.2	5.3
8	Aurel Vlaicu street		43	10.5	33.7	10.6	18.1	32.0	3.2
9			33	11.0	18.6	8.2	12.9	25.8	1.6
